# Validation of the Spanish Version of the Psychological Sense of School Membership (PSSM) Scale in Chilean Adolescents and Its Association with School-Related Outcomes and Substance Use

**DOI:** 10.3389/fpsyg.2016.01901

**Published:** 2016-12-06

**Authors:** Jorge Gaete, Jesus Montero-Marin, Cristian A. Rojas-Barahona, Esterbina Olivares, Ricardo Araya

**Affiliations:** ^1^Department of Epidemiology and Population Health, London School of Hygiene and Tropical MedicineLondon, UK; ^2^Department of Public Health and Epidemiology, Universidad de los AndesSantiago, Chile; ^3^Faculty of Health Sciences and Sports, University of ZaragozaZaragoza, Spain; ^4^Primary Care Prevention and Health Promotion Research Network (RedIAPP)Zaragoza, Spain; ^5^Faculty of Education, Pontificia Universidad Católica de ChileSantiago, Chile; ^6^School of Nursing (Campus San Felipe), Faculty of Medicine, Universidad de ValparaísoSan Felipe, Chile

**Keywords:** validity, school membership, school bonding, substance use, adolescents, truancy

## Abstract

School membership appears to be an important factor in explaining the relationship between students and schools, including school staff. School membership is associated with several school-related outcomes, such as academic performance and expectations. Most studies on school membership have been conducted in developed countries. The Psychological Sense of School Membership (PSSM) scale (18 items: 13 positively worded items, 5 negatively worded items) has been widely used to measure this construct, but no studies regarding its validity and reliability have been conducted in Spanish-speaking Latin American countries. This study investigates the psychometric properties, factor structure and reliability of this scale in a sample of 1250 early adolescents in Chile. Both exploratory and confirmatory factor analyses provide evidence of an excellent fit for a one-factor solution after removing the negatively worded items. The internal consistency of this new abbreviated version was 0.92. The association analyses demonstrated that high school membership was associated with better academic performance, stronger school bonding, a reduced likelihood of school misbehavior, and reduced likelihood of substance use. Analyses showed support for the reliability and validity of the PSSM among Chilean adolescents.

## Introduction

During adolescence, and especially during the early adolescence (ages 10–14), individuals begin to explore their own identity and how they fit in the world. People also begin to project themselves to the future, becoming more distant from their own families (Steinberg and Silverberg, 1986) and relying more on friendships and non-kin relationships for help and guidance (Steinberg and Monahan, 2007).

School is the place where students spend an average of 6 to 7 h every weekday until they complete their secondary education between the ages of 16 to 18. Therefore, school becomes a place where social and contextual influences may have a huge impact on adolescent behaviors. Different variables have been studied to explore the students' connection to school, such as school bonding, school engagement, school involvement, and school membership (Maddox and Prinz, 2003; Libbey, 2004). Each one of them refers to a particular aspect of this relationship. For example, school bonding seems to be a broader concept under which we find the emotional link with the school (school attachment), students' involvement in academic activities (school commitment), students' participation in extra-curricular activities (school involvement), beliefs about school rules (Jenkins, 1997); and school engagement which refers to how much the student engaged in activities related to academic success such as doing homework or achieving better grades (Simons-Morton and Crump, 2003). School membership refers to the students' perceptions on how much teachers and other classmates respect and accept them, and students' feelings of inclusion and belonging. School membership increases engagement in healthy behaviors and reduces the likelihood of engagement in risky behaviors (McNeely and Falci, 2004). High levels of school membership have been associated with better academic motivation and performance (Goodenow and Grady, 1993; Ahn, 2010; Irvin et al., 2011; Moallem, 2013) and better health-promoting behaviors (Gaete et al., 2014). On the contrary, low levels of school membership have been associated with increased negative affect (Shochet et al., 2011), increased suicidality (Stallard et al., 2013), higher school drop-out rates (Finn and Rock, 1997; Ream and Rumberger, 2008), increased school misconduct (Demanet and Van Houtte, 2012), and increased substance use (West et al., 2004). In the last several years, there has been an increasing interest in the evaluation of school-based interventions that aim to improve school climate and school membership; these interventions boast promising results (Bonell et al., 2014; Langford et al., 2014, 2015). However, much less research on this topic has been conducted in Spanish-speaking countries, especially in Latin America. Thus, there is a clear need for a good research instrument in Spanish to measure school membership.

The Psychological Sense of School Membership (PSSM) scale is a self-reported instrument developed to assess the sense of school belonging. The original PSSM scale comprises 18 items: 13 positively worded statements and 5 negatively worded statements. For each statement, students answer on a 5-point scale (1 = not at all true; 5 = completely true). All of these items are related to students' perceptions of being “accepted, respected, included and supported by others in the school social environment” (p. 80)(Goodenow, 1993). It has been widely used—mainly in English-speaking countries—and it has been associated with several variables related to academic achievement, such as increased competence and self-efficacy (Ibañez et al., 2004), increased school attendance (Sánchez et al., 2005), and higher grades (Booker, 2007). Several U.S.-based studies have gathered information from Latino/a or Hispanic students using a Spanish version. However, these studies only report on a few measures of internal reliability (Goodenow, 1993; Robertson et al., 1998; Ibañez et al., 2004; Sánchez et al., 2005; Kuperminc et al., 2008) and do not provide any information about the construct validity or factor structure of the Spanish version.

We have identified eight studies that explored the internal item structure of the 18-item PSSM scale. Three studies were conducted in the U.S. (Hagborg, 1994; O'farrell and Morrison, 2003; Ye and Wallace, 2014); two were conducted in China (Cheung and Hui, 2003; Cheung, 2004); one was conducted in Australia (You et al., 2011); one was conducted in Japan (Togari et al., 2011); and one was a multi-country (Netherland, Kenya, Indonesia, and Spain) comparison study (Abubakar et al., 2015). The latter study included a sample of 14- to 18-year old adolescents from Spain, but some of them responded to the PSSM scale in Spanish while others responded to the scale in Basque (the authors did not provide the exact proportion of respondents for each language) (Abubakar et al., 2015). There is some variability regarding the number of extracted factors that show a good fit, the items included in each factor and the total number of items to be included in the scale. For example, one study found that the school membership scale should include just 5 items from the original scale (O'farrell and Morrison, 2003). A different study found that a one-factor model showed a good fit, using all the items but combining items per target (e.g., school, teachers) (Abubakar et al., 2015). However, two latent factors appear in the Chinese version (Cheung and Hui, 2003; Cheung, 2004), and three factors in other studies (Hagborg, 1994; You et al., 2011). Some studies have suggested that the negatively worded items may constitute a unique factor (Hagborg, 1994; Cheung and Hui, 2003); this does not necessarily imply the existence of a real latent factor in the construct, but rather a methodological issue (Ye and Wallace, 2014). Cultural differences seem to be important when considering a scale in different countries (Abubakar et al., 2015). Noticeably few studies have used a comprehensive data analysis, (i.e., both Exploratory or Confirmatory Factor Analyses) to explore the factor structure of the scale, which may also explain the differences in the findings between the studies (You et al., 2011; Abubakar et al., 2015).

The original purpose of this scale (Goodenow, 1993) was to develop an instrument to assess the social and contextual influences on students' behaviors. The scale was intended to be an instrument with the ability to identify students who are at-risk either for exhibiting risk behaviors and/or dropping out from school. In addition, the scale was initially conceptualized as an unidimensional measure (Goodenow, 1993; You et al., 2011).

The aims of this study are as follows: (1) to explore the factor structure of the Spanish version of the PSSM scale in Chilean early adolescents; (2) to examine the reliability of the resulting scale; and (3) to assess possible relationships between school membership and other school-related outcomes. We hypothesized that school membership will have a positive association with academic variables (academic performance and expectations), and school climate variables (school bonding, school attachment, and school support); conversely, we predicted a negative association between school membership and school misbehavior variables (cheating, truancy, and substance use).

## Materials and methods

### Study design

This is an analytical cross-sectional survey using self-reported information.

### Setting, sample

The population consisted of students in 5th to 8th grade (ages 10–15) from seven schools in San Felipe, a city in the center of Chile. A total of 2108 eligible students were invited to participate; the research team sent a letter to their parents/main caregivers, and informed consent was required to participate. Of those eligible students that were invited, 69.5% consented and participated in the study, resulting in a sample of 1465 students. Completed data for all items of the PSSM scale was gathered from 1250 students. The Ethical Committee at the Faculty of Education of Pontificia Universidad Católica de Chile approved the study protocol. The study followed Helsinki Convention norms.

A qualified nurse (EO) trained several research assistants, who worked in pairs or trios, to administer paper-and-pencil questionnaires to each class. Each school provided a suitable place to conduct the study, assuring that only students with the consent of their parents participated. The study was undertaken in March 2012, the beginning of the academic year in Chile. The day of the survey, research assistants introduced the study to the students, explained the aims and answered questions from the students. All students were asked to sign an informed assent form to participate; no student declined to participate. After the students completed the study, the questionnaires were collected by the research assistants and kept in a sealed envelope to maintain confidentiality.

### Measures

The Psychological Sense of School Membership (PSSM) scale was developed by Goodenow (1993) and consisted of 18 items that measure a perception of membership to the school: 13 positively worded statements intercalated with 5 negatively worded statements. This scale has been used in a large number of studies in different contexts, and it has been translated into several languages (Bernard-Bonnin et al., 2008; Ahn, 2010; Shochet et al., 2011; You et al., 2011; Sari, 2012; Chang et al., 2013; Stallard et al., 2013; Ye and Wallace, 2014; Abubakar et al., 2015; Alkan, 2016). Internal consistency has ranged from 0.73 to 0.95 across samples and countries (Goodenow, 1993; You et al., 2011; Abubakar et al., 2015). The original English version of the scale was received from Carol Goodenow with the authorization to adapt and translate the PSSM to Spanish. Two professional translators produced two Spanish versions. Later, a bilingual expert panel (which included some of the authors) resolved discrepancies from the two Spanish versions, creating one final version. Finally, an independent bilingual translator, whose mother tongue was English, translated the scale back to English, and the expert panel compared the English versions, and a satisfactory final Spanish version was agreed.

#### Sociodemographic variables

Data were collected on age, gender (0 = Male; 1 = Female), grade, school type, religiosity, parental education and occupation, parental marital status, and socioeconomic status (1 = low income; 2 = medium income; 3 = high income). The socioeconomic status was based on the criteria of the 2009 National System for the Measurement of Education Quality (Ministry of Education (Chile), 2010), which gathers information from parents or main caregivers about their household income.

#### School variables

In order to explore the relationship between school membership and other school-related factors, we collected the following data:

#### Self-reported academic performance

We collected each student's grade point average (GPA) from the previous school year. The Chilean GPA scale ranges from 1 to 7, and the approval cutoff for passing is 4.0. We asked students to select their GPA from one of the following categories: 1 = Below 4.0; 2 = Between 4.1 and 4.4; 3 = Between 4.5 and 4.9; 4 = Between 5.0 and 5.4; 5 = Between 5.5 and 5.9; 6 = Between 6.0 and 6.4; 7 = Between 6.5 and 7.0.

#### Academic expectations

We asked if students were concerned about failing in school this year on a 4-point scale (1 = not at all concerned to 4 = extremely concerned). This item is part of a questionnaire of adolescent health that has been validated in Chile (Blum et al., 1988; Florenzano et al., 1993).

#### School bonding

We used a scale similar to the one used by Hawkins et al. (2001) to assess school bonding. It included the following items: “I like school,” “Most mornings I look forward to going to school,” “I do extra school work on my own,” “When I have an assignment to do, I keep working on it until it is finished,” and “I like my classes this year.” Response options for each item were as follows: 1 = NO!; 2 = no; 3 = yes; and 4 = YES!. Each student's responses to the 5 items were averaged together to create their total score. A higher score indicates a higher level of school bonding. The Cronbach's alpha of this scale in our sample was 0.77. This scale measures concepts similar to school bonding, such as school attachment and school commitment, following Maddox and Printz's conceptualization (Maddox and Prinz, 2003). Therefore, the construct is similar to school membership, but not quite the same. We followed a similar procedure to obtain the Spanish version of this scale as the procedure we followed with the PSSM scale.

#### School liking

We included a single item exploring the concept of school attachment: “How do you feel about your school?” Response options were: 1 = I hate my school; 2 = I don't like it; 3 = I like it somewhat; and 4 = I like it very much. This item is part of a questionnaire of adolescent health that has been validated in Chile (Blum et al., 1988; Florenzano et al., 1993).

#### School staff support

We asked students if they believe that school staff (e.g., school staff, administrator, teachers) care about them. Response options were: 1 = not at all; 2 = somewhat; 3 = very much. This item is part of a questionnaire of adolescent health that has been validated in Chile (Blum et al., 1988; Florenzano et al., 1993).

#### School misbehavior

We assessed school misbehavior using two items. The first item was about cheating: “During the last year, how many times did you copy answers from others?” The second item was about truancy: “During the last month, how many days did you skip classes or did not attend school without permission?” Students responded to each item using a 5-point scale (1 = never to 5 = more than 10 times). This item is part of a questionnaire of adolescent health that has been validated in Chile (Blum et al., 1988; Florenzano et al., 1993).

#### Substance use

We asked students about their use of tobacco, alcohol, marijuana, inhalants, stimulants, cocaine, and other drugs during the past year. We operationalized this construct as a binary variable (0 = no usage of any drug in the last year; 1 = usage of some drug in the last year).

### Data analysis

All analyses were performed using STATA 12.1, Factor 9.02, and Amos 7. Firstly, we examined the sociodemographic variables, as well as the items' psychometric characteristics by using descriptive statistics (i.e., means, standard deviations, frequencies, percentages, skewness, and kurtosis).

#### Dimensionality

To analyze the factor structure, we randomly split the data into two halves: The first half (*n*_1_ = 625) was used for the initial Exploratory Factor Analysis (EFA), and the second half (*n*_2_ = 625) was used for the Confirmatory Factor Analysis (CFA). We estimated Mardia's coefficients (Mardia, 1974) to assess the multivariate distribution of the variables. Polychoric correlations are advised for factor analysis when the distributions of ordinal items are asymmetric with excess of kurtosis or high item-total correlations (Muthen and Kaplan, 1992). For each sub-sample, a polychoric correlation matrix of PSSM scale items was estimated, and the unweighted least squares factor analysis (ULS) was used for factor extraction, due to its robustness (Jöreskog, 1977). This procedure does not require any distributional assumptions; it usually converges because of its efficiency in terms of computation; it tends to provide less biased estimates of the true parameter value; and it works well with polychoric matrices (Briggs and Maccallum, 2003). We firstly observed the unrotated solution of the EFA, but we also used the promin rotation method to facilitate interpretations, given the correlated solution expected (Lorenzo-Seva, 1999).

We used parallel analysis (Horn, 1965) to identify the number of factors to include in the solution. This identification was performed by replacing the raw data method with optimal implementation based on minimum rank factor analysis (Timmerman and Lorenzo-Seva, 2011), generating 500 random correlation matrices. With this analysis, a factor is significant if the associated eigenvalue is larger than that corresponding to a given percentile, such as the 95th percentile of the distribution of eigenvalues derived from a random dataset. This method is the best available solution to decide the number of factors to retain for a given scale (Hayton et al., 2004; Ledesma and Valero-Mora, 2007). To select the items to be included in each latent factor, we used the criterion of factorial loading (i.e., <0.5) (Comrey and Lee, 2013). If any item loaded lower than 0.5, we removed those items, then we conducted the EFA again, in an iterative way, repeating this procedure until all the items loaded higher than 0.5 in their corresponding factor (i.e., until they showed a clear and simple structure of weightings). Factor weights (w) and the percentage of explained variance in each item by means of communality values (*h*^2^) were calculated. For each EFA, we tested the goodness of fit of the exploratory model using the goodness of fit index (GFI) (Maiti and Mukherjee, 1990) and the root mean square of residuals (RMSR) (Harman, 1962).

Using CFA and by applying ULS from the second polychoric matrix, we examined the goodness of fit of the proposed PSSM scale. From a general perspective, we used the goodness of fit index (GFI), the adjusted goodness of fit index (AGFI), the root mean square of the standardized residuals (RMSR), the normed-fit-index (NFI), and Bollen's relative-fit-index (RFI). GFI and AGFI refer to explained variance; values greater than 0.90 are considered acceptable (Byrne, 2001). SRMR is the standardized difference between the observed and the predicted covariance; values lower than 0.08 indicate a good fit (Hu and Bentler, 1999). NFI measures the proportional reduction in the adjustment function when going from null to the proposed model; values greater than 0.90 are considered acceptable (Lévy et al., 2006). RFI takes into account the discrepancy for the proposed model and for the baseline model; values that are close to 1 indicate a good fit (Bollen, 1986). Standardized saturations and the proportion of explained variance were also examined.

#### Reliability

We examined the reliability of the scale using congeneric, tau-equivalent and parallel models for the whole sample (*n* = 1250), using the ULS method. The three models assume that all items measure the same latent factor. However, the congeneric model is the least restrictive model, with potentially different scales, degrees of precision, and magnitude of error. The tau-equivalent implies that items are on the same scale, with the same degree of precision, but with possibly different degrees of error. The parallel model is the most restrictive, where all items are on the same scale, with the same degree of precision, and with the same amount of error (Raykov, 1997). The reliability value was estimated by squaring the implied correlation between the composite latent true variable and the composite observed variable, to arrive at the percentage of the total observed variance that was accounted for by the “true” variable (Graham, 2006). The mean of item-total values (discrimination coefficients) was also assessed.

#### Associations

We explored the associations between school membership (as an independent variable) and the school-related factors (as dependent variables) using regression models and controlling for age, gender, and socioeconomic status. In most cases, the dependent variable was operationalized as a continuous variable; therefore, simple regression models were used. Standardized slopes (beta) and coefficients of determination (*R*^2^) were also calculated. For the case of substance use (binary variable), a logistic regression was performed, and odds ratios were examined. The cutoff for statistical significance was established at *p* < 0.05 and 95% confidence intervals were calculated and presented.

## Results

### Description of participants

Table 1 shows the general features of the total sample and for each sub-sample. The sample consisted of students between the ages of 10–15 (mean = 11.9 years: *SD* = 1.2 years), and 45% were girls. Most of the students came from low socioeconomic status (45%) and there was a fairly similar distribution of grade level (20.5–27.4%). Nearly one-fifth (18.3%) of the students considered themselves as very religious. Most students lived with both parents (63.8%); further, over half the parents completed secondary education (58.7% of mothers and 54.2% of fathers). Almost 70% of fathers worked full-time, while 44.1% of mothers worked full-time.

**Table 1:** **Characteristics of participants in total sample (*N* = 1250), and in the sub-samples used (*n*_1_ = 625; *n*_2_ = 625)**.

**Variable**	**Total *N* [Mean (*SD*)]/[%(95%CI)]**	**Sub-sample 1 *n*_1_ [Mean (*SD*)]/[%(95%CI)]**	**Sub-sample 2 *n*_2_ [Mean (*SD*)]/[%(95%CI)]**
Age (10–15)	1250 [11.9 (1.2)]	625 [12.0 (1.2)]	625 [11.9 (1.2)]
Sex, [females]	561 [45.0 (42.2–47.7)]	279 [44.7 (40.8–48.6)]	282 [45.2 (41.3–49.1)]
**SOCIOECONOMIC LEVEL**			
Low	563 [45.0 (42.3–47.8)]	292 [46.7 (42.–50.6)]	271 [43.4 (39.5–47.3)]
Medium	405 [32.4 (29.8–35.0)]	199 [31.8 (28.1–35.5)]	206 [33.0 (29.3–36.7)]
High	282 [22.6 (20.2–24.9)]	134 [21.4 (18.2–24.7)]	148 [23.7(20.3–27.0)]
**GRADE**			
Year 5	342 [27.4 (24.9–29.8)]	176 [28.2 (24.6–31.7)]	166 [26.6 (23.1–30.3)]
Year 6	326 [26.1 (23.6–28.5)]	161 [25.8 (22.3–29.2)]	165 [26.4 (22.9–29.9)]
Year 7	326 [26.1 (23.6–28.5)]	159 [25.4 (22.0–28.9)]	167 [26.7 (23.2–30.2)]
Year 8	256 [20.5 (18.2–22.7)]	129 [20.6 (17.5–23.8)]	127 [20.3 (17.2–23.5)]
**GRADE POINT AVERAGE (1 TO 7)**			
<4.0	11 [0.9 (0.4–1.4)]	4 [0.6 (0.0–1.3)]	7 [1.1 (0.3–2.0)]
4.0–4.4	31 [2.5 (1.6–3.4)]	18 [2.9 (1.6–4.3)]	13 [2.1 (1.0–3.3)]
4.5–4.9	92 [7.5 (6.0–9.0)]	55 [8.9 (6.7–11.2)]	37 [6.0 (4.1–7.9)]
5.0–5.4	195 [15.9 (13.8–17.9)]	98 [15.9 (13.0–18.8)]	97 [15.8 (12.9–18.7)]
5.5–5.9	348 [28.3 (25.8–30.8)]	171 [27.8 (24.2–31.3)]	177 [28.8 (25.2–32.4)]
6.0–6.4	353 [28.7 (26.2–31.2)]	166 [26.9 (23.4–30.5)]	187 [30.5 (26.8–34.1)]
6.5–7.0	200 [16.3 (14.2–18.3)]	104 [16.9 (13.9–19.8)]	96 [15.6 (12.8–18.5)]
**RELIGIOSITY**			
Very religious	224 [18.3 (16.1–20.4)]	112 [18.3 (15.2–21.3)]	112 [18.2 (15.2–21.3)]
Somewhat religious	847 [69.0 (66.4–71.6)]	427 [69.6 (66.0–73.3)]	420 [68.4 (64.7–72.1)]
Not at all religious	156 [12.7 (10.8–14.6)]	74 [12.1 (9.5–14.6)]	82 [13.4 (10.7–16.1)]
**PARENTAL MARITAL STATUS**			
Married and living together	568 [45.6 (42.9–48.4)]	289 [46.5 (42.6–50.5)]	279 [44.7 (40.8–48.6)]
Never married but living together	226 [18.2 (16.0–20.3)]	107 [17.2 (14.3–20.2)]	119 [19.1 (16.0–22.2)]
Married but living apart	198 [15.9 (13.9–17.9)]	100 [16.1 (13.2–19.0)]	98 [15.7 (12.8–18.6)]
Divorced	58 [4.7 (3.5–5.8)]	29 [4.7 (3.0–6.3)]	29 [4.6 (3.0–6.3)]
Never married and living apart	131 [10.5 (8.8–12.2)]	61 [9.8 (7.5–12.2)]	70 [11.2 (8.7–13.7)]
One or both parents are dead	19 [1.5 (0.8–2.2)]	10 [1.6 (0.6–2.6)]	9 [1.4 (0.5–2.4)]
Do not know	45 [3.6 (2.6–4.7)]	25 [4.0 (2.5–5.6)]	20 [3.2 (1.8–4.6)]
**MOTHER's EDUCATION**			
No education	7 [0.6 (0.1–1.0)]	5 [0.8 (0.1–1.5)]	2 [0.3 (–0.1–0.8)]
Primary unfinished	91 [7.4 (5.9–8.8)]	49 [7.9 (5.8–10.1)]	42 [6.8 (4.8–8.8)]
Primary finished	118 [9.6 (7.9–11.2)]	61 [9.9 (7.5–12.2)]	57 [9.2 (6.9–11.5)]
Secondary unfinished	157 [12.7 (10.9–14.6)]	77 [12.5 (9.8–15.1)]	80 [13.0 (10.3–15.6)]
Secondary finished	352 [28.5 (26.0–31.0)]	172 [27.8 (24.3–31.4)]	180 [29.1 (25.6–32.8)]
Higher education unfinished	43 [3.5 (2.5–4.5)]	17 [2.8 (1.5–4.4)]	26 [4.2 (2.6–5.8)]
Higher education finished	229 [18.5 (16.4–20.7)]	124 [20.1 (16.9–23.2)]	105 [17.0 (14.0–20.0)]
Do not know	235 [19.0 (16.8–21.2)]	112 [18.1 (15.1–21.2)]	123 [19.9 (16.8–23.1)]
Others	3 [0.2 (–0.0–0.5)]	1 [0.2 (–0.2–0.5)]	2 [0.3 (–0.1–0.8)]
**MOTHER's OCCUPATION**			
Working full-time	536 [44.1 (41.3–46.9)]	257 [42.5 (38.5–46.4)]	279 [45.7 (41.8–49.7)]
Working part-time	290 [23.9 (21.5–26.3)]	145 [24.0 (20.6–27.4)]	145 [23.8 (20.4–27.2)]
Unemployed	74 [6.1 (4.7–7.4)]	34 [5.6 (3.8–7.5)]	40 [6.6 (4.6–8.5)]
Retired	3 [0.3 (–0.0–0.5)]	1 [0.2 (–0.2–0.5)]	2 [0.3 (–0.1–0.8)]
Disabled-unable to work	32 [2.6 (1.7–3.5)]	18 [3.0 (1.6–4.3)]	14 [2.3 (1.1–3.5)]
Other	280 [23.1 (20.7–25.4)]	150 [24.8 (21.3–28.2)]	130 [21.3 (18.1–24.6)]
**FATHER's EDUCATION**			
No education	18 [1.5 (0.8–2.1)]	9 [1.5 (0.5–2.4)]	9 [1.5 (0.5–2.4)]
Primary unfinished	86 [7.0 (5.6–8.4)]	41 [6.7 (4.7–8.6)]	45 [7.3 (5.3–9.4)]
Primary finished	102 [8.3 (6.7–9.8)]	61 [9.9 (7.5–12.3)]	41 [6.7 (4.7–8.6)]
Secondary unfinished	135 [11.0 (9.2–12.7)]	65 [10.6 (8.1–13.0)]	70 [11.4 (8.9–13.9)]
Secondary finished	326 [26.5 (24.0–29.0)]	171 [27.8 (24.2–31.3)]	155 [25.2 (21.8–28.6)]
Higher education unfinished	31 [2.5 (1.6–3.4)]	23 [3.7 (2.2–5.2)]	8 [1.3 (0.4–2.2)]
Higher education finished	216 [17.6 (15.4–19.7)]	102 [16.6 (13.6–19.5)]	114 [18.5 (15.5–21.6)]
Do not know	315 [25.6 (23.1–28.0)]	144 [23.4 (20.0–26.7)]	171 [27.8 (24.3–31.4)]
Others	2 [0.2 (–0.0–0.4)]	0	2 [0.3 (–0.1–0.8)]
**FATHER's OCCUPATION**			
Working full-time	843 [69.2 (66.7–71.8)]	425 [69.6 (65.9–73.2)]	418 [68.9 (65.2–72.6)]
Working part-time	251 [20.6 (18.3–22.9)]	131 [21.4 (18.2–24.7)]	120 [19.8 (16.6–22.9)]
Unemployed	25 [2.1 (1.3–2.8)]	11 [1.8 (0.7–2.9)]	14 [2.3 (1.1–3.5)]
Retired	9 [0.7 (0.3–1.2)]	3 [0.5 (–0.1–1.0)]	6 [1.0 (0.2–1.8)]
Disabled-unable to work	12 [1.0 (0.4–1.5)]	3 [0.5 (–0.1–1.0)]	9 [1.5 (0.5–2.4)]
Other	78 [6.4 (5.0–7.8)]	38 [6.2 (4.3–8.1)]	40 [6.6 (4.6–8.6)]

### Descriptives and dimensionality

The polychoric matrix of the PSSM items using the first sub-sample revealed that 52.9% of the coefficients on the diagonal were greater than 0.30. The determinant of the matrix was 0.003. KMO test had a value of 0.90 (very good) and Bartlett's statistic was 3841.7 (df = 153; *p* < 0.001). Table 2 shows the descriptive statistics of all 18 items and the loading factors for each step of the iterative EFA that was explained in the methods section. The unrotated solution was difficult to interpret in terms of meaning, so that we relied on the promin rotation to facilitate this task.

**Table 2:** **Iterative process of discarding inadequate PSSM scale items through Exploratory Factor Analysis (*n*_1_ = 625)**.

**Items**	**Md**	***SD***	**skew**	**kurt**	**1st EFA**		**2nd EFA**	**3rd EFA**	
					**F_1_**	**F_2_**	**F_1_**	**F_1_**	***h*^2^**
1. I felt like a real part of (name of school)	2.45	1.37	0.61	−0.84	0.63	0.19	0.58	0.67	0.45
2. People notice when I'm good at something	2.94	1.41	0.13	−1.27	0.68	0.21	0.62	0.66	0.44
3. It is hard for people like me to be accepted here [reversed]^*^	1.99	1.42	1.16	−0.14	0.26	0.43	–	–	–
4. Other students in this school take my opinions seriously	2.98	1.45	0.10	−1.36	0.71	0.06	0.70	0.69	0.47
5. Most teachers at this school are interested in me	3.11	1.45	0.01	−1.37	0.69	0.03	0.69	0.73	0.53
6. Sometimes I don't feel as if I belong here. [reversed]^**^	1.86	1.26	1.38	0.71	0.09	0.67	−0.06	–	–
7. There's at least one teacher or other adult in this school I can talk to if I have a problem	3.03	1.65	0.01	−1.65	0.59	0.10	0.57	0.60	0.35
8. People at this school are friendly to me	3.47	1.43	−0.35	−1.29	0.73	−0.10	0.75	0.74	0.55
9. Teachers here are not interested in people like me. [reversed]^**^	1.86	1.24	1.33	0.63	0.07	0.56	−0.06	–	–
10. I am included in lots of activities at this school	2.91	1.52	0.17	−1.43	0.58	0.25	0.51	0.59	0.34
11. I am treated with as much respect as other students	3.73	1.46	−0.70	−1.02	0.78	−0.07	0.80	0.74	0.55
12. I feel very different from most other students here. [reversed]^*^	2.24	1.51	0.82	−0.86	0.19	0.48	–	–	–
13. I can really be myself at this school	3.64	1.57	−0.60	−1.27	0.67	0.03	0.67	0.66	0.43
14. The teachers here respect me	3.98	1.38	−0.98	−0.55	0.73	−0.09	0.75	0.78	0.61
15. People here know I can do good work	3.82	1.42	−0.83	−0.79	0.75	−0.08	0.76	0.79	0.63
16. I wish I were in a different school. [reversed]^*^	1.83	1.40	1.46	0.54	−0.06	0.48	–	–	–
17. I feel proud of belonging to (name of school)	3.82	1.52	−0.79	−0.99	0.70	0.23	0.74	0.69	0.47
18. Other students here like me the way I am	3.48	1.43	−0.44	−1.18	0.68	−0.01	0.68	0.68	0.46

*Md, median; SD, standard deviation; skew, skewness; kurt, kurtosis; F_1_, first factor; F_2_, second factor; 1st EFA, first Exploratory Factor Analysis using the total PSSM questionnaire (with a solution of two factors by using parallel analysis); 2nd EFA, second Exploratory Factor Analysis using the PSSM questionnaire without the discarded items (w < 0.5) in the previous 1st EFA (with a solution of one factor using parallel analysis); 3rd EFA, third Exploratory Factor Analysis using the PSSM questionnaire without the discarded items (w < 0.5) in the previous 1st and 2nd EFAs (with a solution of one factor using parallel analysis)*;

* *, item discarded as a result of the first EFA*;

** *, item discarded as a result of the second EFA; h^2^, communality of the finally selected PSSM items*.

We needed to conduct the EFA three times. In the first EFA, we observed two factors that explained 40.9% (F_1_) and 13.3% (F_2_) of the variance (GFI = 0.95; RMSR = 0.10). Most items loaded onto F_1_; only two items (items 6 and 9) loaded onto F_2_. Three other items (items 3, 12, and 16) did not load onto any of the factors over 0.5, so they were removed. When we conducted the second EFA with 15 items, only one factor appeared; this factor explained 51.6% of the variance (GFI = 0.98; RMSR = 0.07). In this factor, all items but two (6 and 9) had a factor loading greater than 0.5; therefore, items 6 and 9 were removed. Next, we conducted a third EFA with 13 items, finding one factor that explained 54.2% of the variance, (GFI = 0.99; RMSR = 0.06). Finally, all items had a factor loading greater than 0.5. Thus, the best fit was found in the latter exploratory factor model.

The polychoric matrix of the PSSM scale items using the sub-sample 2 revealed that 100% of the coefficients on the diagonal were over 0.30. The determinant of the matrix was 0.005. The KMO test had a value of 0.94 (very good) and Bartlett's statistic was 3167.4 (df = 783; *p* < 0.001). Table 3 shows the descriptive statistics of the 13-item scale and the PSSM one-factor structure using CFA from both an analytical and standardized point of view. The factor loadings for the items were very high (ranges from 0.59 to 0.79). This structure possessed adequate fit indices (Table 4), with no correlations between the error terms (GFI = 0.99; AGFI = 0.99; NFI = 0.99; RFI = 0.99; RMSR = 0.04).

**Table 3:** **Descriptive statistics for items selected for CFA (*n*_2_ = 625)**.

**Items**	**Mean**	***SD***	**Skewness**	**Kurtosis**	**Weights**	***h*^2^**
1. I felt like a real part of (name of school)	2.39	1.40	0.66	−0.87	0.67	0.45
2. People notice when I'm good at something	2.94	1.40	0.15	−1.24	0.66	0.43
4. Other students in this school take my opinions seriously	3.05	1.44	0.04	−1.34	0.69	0.47
5. Most teachers at this school are interested in me	3.00	1.47	0.11	−1.41	0.73	0.53
7. There's at least one teacher or other adult in this school I can talk (…)	2.96	1.65	0.09	−1.63	0.59	0.35
8. People at this school are friendly to me	3.49	1.39	−0.33	−1.25	0.74	0.55
10. I am included in lots of activities at this school	3.03	1.45	0.09	−1.36	0.59	0.34
11. I am treated with as much respect as other students	3.63	1.50	−0.57	−1.22	0.74	0.55
13. I can really be myself at this school	3.56	1.58	−0.52	−1.35	0.66	0.43
14. The teachers here respect me	3.90	1.41	−0.89	−0.72	0.78	0.61
15. People here know I can do good work	3.77	1.38	−0.71	−0.87	0.79	0.63
17. I feel proud of belonging to (name of school)	3.77	1.49	−0.70	−1.11	0.68	0.47
18. Other students here like me the way I am	3.34	1.40	−0.23	−1.27	0.68	0.46

**Table 4:** **Adjustment indices for CFA and models of reliability of the PSSM scale**.

	**R**	**GFI**	**AGFI**	**RSMR**	**NFI**	**RFI**
Structural model		0.99	0.99	0.04	0.99	0.99
Congeneric model of reliability^*^	0.92	0.99	0.99	0.05	0.99	0.99
Tau-equivalent model of reliability^*^	0.92	0.99	0.98	0.07	0.98	0.98
Parallel model of reliability^*^	0.92	0.98	0.98	0.07	0.98	0.98

*R, Reliability; GFI, Goodness of Fit Index; RSMR, Root Mean Square of the Standardized Residuals; AGFI, Adjusted Goodness of Fit Index; NFI, Normed Fit Index; RFI, Relative Fit Index*.

* *, total sample (n_t_ = 1250)*.

### Reliability

Table 4 shows the reliability models tested for the PSSM structure. As it can be seen, the best fit indices were for the congeneric model, although the parallel model also indicates a good fit for the data (GFI = 0.92; AGFI = 0.98; NFI = 0.98; RFI = 0.98; RMSR = 0.07). The reliability values were very good in all the models (*R* = 0.92). The mean of the item-total values was 0.60 (range: 0.49 to 0.66).

### Associations

We found that school membership was significantly associated with all measured school-related factors, controlling for age, gender and socioeconomic status, although the effect size varied considerably across variables. Specifically, a higher score in school membership was associated with higher levels of school performance (β = 0.29; *p* < 0.001), school bonding (β = 1.60; *p* < 0.001), school attachment (β = 0.21; *p* < 0.001); school expectations (β = 0.11; *p* < 0.001), and school staff support (β = 0.29; *p* < 0.001). A higher score in school membership was also associated with lower levels of cheating (β = −0.12; *p* < 0.001), and truancy (β = −0.04; *p* = 0.007). We also found that a high score in school membership predicted a reduced risk of having used any substance during the last 12 months (OR = 0.60; *p* = 0.010). Effect sizes were high for school bonding (*R*^2^ = 0.29), being moderate for school performance (*R*^2^ = 0.18), school support (*R*^2^ = 0.16), cheating (*R*^2^ = 0.14), and school attachment (*R*^2^ = 0.11), and low for substance use (*R*^2^ = 0.05), school expectations (*R*^2^ = 0.02), and truancy (*R*^2^ = 0.01). Additionally, we found that older students tended to have lower grades, lower school bonding, lower school attachment, and higher risk for cheating. Females, compared to their male counterparts, had higher grades but also reported more cheating. Higher socioeconomic status was related to higher school performance and lower likelihood cheating (Table 5).

**Table 5:** **Regression models of the association between school membership and school-related factors; controlling for age, gender, and socioeconomic status (SES)**.

**Variable**	**GPA**		**School bonding**		**School attachment**		**School expectations**		**School support**		**Cheating**		**Truancy**		**Substance use**	
	***B* (95%CI)**	***R*^2^**	***B* (95%CI)**	***R*^2^**	***B* (95%CI)**	***R*^2^**	***B* (95%CI)**	***R*^2^**	***B* (95%CI)**	***R*^2^**	***B* (95%CI)**	***R2***	***B* (95%CI)**	***R*^2^**	***OR^a^*(95%CI)**	***R*^2^^b^**
School membership	0.29^**^ (0.22 to 0.36)	0.18	0.32^**^ (0.29 to 0.35)	0.29	0.21^**^ (0.17 to 0.24)	0.11	0.11^**^ (0.07 to 0.16)	0.02	0.29^**^ (0.25 to 0.32)	0.16	−0.12^**^ (−0.17 to −0.07)	0.14	−0.04^*^ (0.07 to −0.01)	0.01	0.60^*^ 0.41 to 0.89	0.05
Age	−0.31^**^ (−0.36 to −0.25)		−0.14^**^ (−0.17 to −0.11)		−0.05^*^ (−0.08 to 0.02)		-0.01 (−0.04 to 0.02)		0.02 (−0.01 to 0.04)		0.22^**^ (0.17 to 0.26)		0.00 (−0.02 to 0.02)		1.11 (0.82 to 1.49)	
Sex	0.14^*^ (0.00 to 0.28)		0.03 (−0.04 to 0.10)		0.04 (−0.03 to 0.11)		0.03 (−0.05 to 0.11)		0.07 (−0.01 to 0.14)		0.15^*^ (0.04 to 0.25)		−0.01 (−0.06 to 0.04)		0.51 (0.22 to 1.17)	
SES	0.18^**^ (0.09 to 0.26)		−0.04^*^ (−0.08 to −0.00)		0.03 (−0.02 to 0.07)		−0.01 (0.06 to 0.04)		0.01 (−0.04 to 0.06)		−0.22^**^ (−0.29 to −0.16)		−0.01 (−0.05 to 0.02)		0.77 (0.45 to 1.30)	

* *p < 0.05*;

** *p < 0.001*;

a *OR, odds ratio*;

b *Pseudo R^2^*.

## Discussion

We found that the Spanish version of the PSSM scale measures one underlying factor, *sense of school belonging*. However, our analyses suggested that not all items performed well, particularly those that were negatively worded. We found that when using all 18 items, the negatively worded items may have created a methodological artifact—one that has already been found in some studies exploring the same scale (Ye and Wallace, 2014; Abubakar et al., 2015) or in studies using other scales (Marsh, 1996; Hankins, 2008). However, when removing all negatively worded items, the structure and the loading of the items in the scale appear to be consistent. Therefore, after the EFA analyses, one latent factor was found, and the CFA of these items results in excellent goodness of fit indices.

Several authors have found different item-structures of this scale. Initially, Goodenow did not report any analysis of the structure of the scale, but she was using it as unidimensional scale with good reliability (Goodenow, 1993). Later, using a small sample, Hagborg identified three factors, and one of them was labeled as “*belonging*” which consisted of 11 items (Hagborg, 1994), all of which were positively worded. In a subsequent study, Hagborg used this abbreviated scale of 11 items and obtained good results, supporting the idea that the PSSM scale should be considered unidimensional. Nonetheless, other studies have identified more than one latent factor; however, in many cases, these factors did not include the same items. Two recent studies, each with a good sample size (You et al., 2011; Ye and Wallace, 2014), used a combination of EFA and CFA conducted in different samples; the results suggested that there were three latent factors. However, the items that loaded onto particular factors were different across the two studies.

We noted that Cheung and Hui's study found 2 underlying factors, one with the 13 positively-worded items and another with the 5 negatively worded items (Cheung and Hui, 2003). In You et al. (2011) after removing six items because of cross-loading, three latent factors were identified: (1) caring relationships with teachers (4 items: 3 positively worded and 1 negatively worded); (2) acceptance (5 positively worded items); and (3) rejection (three negatively worded items). Taking this information into account, Ye and Wallace (2014) conducted a survey with U.S. secondary students from ages 13 to 19. The researchers explored whether a method effect caused previous studies to identify a factor including only negatively worded items, or if there is more than one substantive factor in the scale (Ye and Wallace, 2014). The authors found that there was a method effect related to the wording of the items and they advised that further research needed to explore the implications of this effect. However, the researchers removed 3 items, and they decided to keep this factor in the scale, thus proposing a structure solution with 3 factors: (1) identification with and participation in the school; (2) perception of fitting in among peers; and (3) generalized connections to teachers.

Finally, the most recent published study gathered information from four countries (Abubakar et al., 2015); in one of those countries, a Spanish version of the scale was used. The authors explored several models for the relationship between items, and they also studied the potential methodological artifact introduced by the negatively-worded items. These researchers found evidence that the PSSM scale has a unidimensional structure, and they supported the idea that the negatively-worded items were not producing an artificial multidimensional structure that was found in other studies. The researchers also posited that it is possible to consider the items as forming three sub-scales according to the targets or contexts to which the items were referring (Abubakar et al., 2015). The authors did not propose removing any items but instead considered that there may be a need to refine the scale further to ensure clarity in measurement. Our study differed from this one because the adolescents in their study were older (ages 14–18) and they also had a smaller sample (*n* = 590).

In our study, the first examination of the psychometric properties of the PSSM scale in Latin American early adolescents, we found that the negatively worded items did not load into the main factor or into any other factor. Furthermore, the latent factor uncovered in our study was very similar to the one originally found by Hagborg (1994). One potential explanation for our results may be related to the age range of our sample. It is possible that, due to the cognitive development and proficiency in reading comprehension, students who were ages 10–14 might have had difficulty understanding the answer format or the direction of the intercalated questions. Many of the studies reviewed here explored the psychometric properties of the scale with older adolescents (Abubakar et al., 2015). However, in a study with 4–6th graders in the U.S., the negatively-worded items appeared to load onto a separate factor than the positively worded items (O'farrell and Morrison, 2003), giving some support to the idea that there could be a methodological artifact.

The reliability of the final proposed items to form the PSSM scale was very high (0.92), which could be considered one of the highest internal consistency obtained so far (You et al., 2011). Moreover, each item seemed to be measuring the corresponding latent variable with possibly the same degree of precision and the same amount of error. Therefore, we propose the use of an abbreviated version of the PSSM scale, which includes only the positively worded items (13 items), to allow for the assessment of school membership among adolescents (ages 10–19) (Sawyer et al., 2012) and we encourage the comparison of the results across cultures.

The exploration of the associations between the sense of school membership and other relevant school-related outcomes supports the idea that school membership seems to be a good measure of the relationship between students and schools, either as a predictor of academic outcomes (achievement, truancy, drop-out), as a potential mediator for interventions aiming to increase school climate or even to improve misbehavior. It is important to continue studying this construct to better understand potential changes over time while students get older and to determine how they respond to different interventions.

One limitation of this study is related to its cross-sectional design, which makes it difficult to establish a longitudinal and truly predictive value of this measure. Another limitation is the use of retrospective, self-reported measures in an adolescent population, which could have influenced the accuracy of the reporting. For instance, cognitive factors might introduce reporting bias (related to comprehension of questions and decision-making issues) (Pokorski et al., 1994) or retrieval errors (especially for long periods of time)(Bachman and O'malley, 1981). Situational factors that affect the self-reporting may include students' perceptions of the confidentiality of the information (Hedges and Jarvis, 1998) or social desirability (Brittingham et al., 1998). When administering the PSSM scale, research assistants did not report any complaints about the comprehension of the items. Regarding the rest of the questionnaire, many of the questions have been used in other studies in Chile for many years (Senda, 2014) without any reported problems. Regarding the situational factors, numerous measures were taken out to ensure anonymity and confidentiality. Furthermore, there is evidence some that the cognitive and situational factors mentioned above do not threaten the validity of self-reported measurements among students (Brener et al., 2003).

## Conclusions

The Psychological Sense of School Membership scale (PSSM), using 13 of the 18 original items, appears to have a good structure, validity, and a high reliability when used to assess Latin American adolescent students. We have provided evidence that this seems to be a unidimensional scale, and using only the positively worded items could provide a clearer solution among adolescents of a wide age range.

## Author contributions

JG, CR, and EO conceived and designed the study and supervised the collection of data. JG and JM analyzed and interpreted the data, and produced the drafting of the manuscripts. RA supervised all steps in the study. All authors provided a critical revision of the manuscript.

### Conflict of interest statement

The authors declare that the research was conducted in the absence of any commercial or financial relationships that could be construed as a potential conflict of interest.
